# Electrophysiological correlates of the BOLD signal for EEG‐informed fMRI

**DOI:** 10.1002/hbm.22623

**Published:** 2014-10-03

**Authors:** Teresa Murta, Marco Leite, David W. Carmichael, Patrícia Figueiredo, Louis Lemieux

**Affiliations:** ^1^ Department of Clinical and Experimental Epilepsy UCL Institute of Neurology, Queen Square London United Kingdom; ^2^ Department of Bioengineering Institute for systems and robotics, Instituto Superior Técnico, Universidade de Lisboa Lisbon Portugal; ^3^ Imaging and Biophysics Unit UCL Institute of Child Health London United Kingdom; ^4^ MRI Unit, Epilepsy Society Chalfont St. Peter United Kingdom

**Keywords:** electrophysiology, functional magnetic resonance imaging, correlation, human brain, coupling

## Abstract

Electroencephalography (EEG) and functional magnetic resonance imaging (fMRI) are important tools in cognitive and clinical neuroscience. Combined EEG–fMRI has been shown to help to characterise brain networks involved in epileptic activity, as well as in different sensory, motor and cognitive functions. A good understanding of the electrophysiological correlates of the blood oxygen level‐dependent (BOLD) signal is necessary to interpret fMRI maps, particularly when obtained in combination with EEG. We review the current understanding of electrophysiological–haemodynamic correlates, during different types of brain activity. We start by describing the basic mechanisms underlying EEG and BOLD signals and proceed by reviewing EEG‐informed fMRI studies using fMRI to map specific EEG phenomena over the entire brain (EEG–fMRI mapping), or exploring a range of EEG‐derived quantities to determine which best explain colocalised BOLD fluctuations (local EEG–fMRI coupling). While reviewing studies of different forms of brain activity (epileptic and nonepileptic spontaneous activity; cognitive, sensory and motor functions), a significant attention is given to epilepsy because the investigation of its haemodynamic correlates is the most common application of EEG‐informed fMRI. Our review is focused on EEG‐informed fMRI, an asymmetric approach of data integration. We give special attention to the invasiveness of electrophysiological measurements and the simultaneity of multimodal acquisitions because these methodological aspects determine the nature of the conclusions that can be drawn from EEG‐informed fMRI studies. We emphasise the advantages of, and need for, simultaneous intracranial EEG–fMRI studies in humans, which recently became available and hold great potential to improve our understanding of the electrophysiological correlates of BOLD fluctuations. *Hum Brain Mapp, 36:391–414, 2015*. © **2014 The Authors Human Brain Mapping Published by Wiley Periodicals, Inc.**

## INTRODUCTION

**Figure 1:**
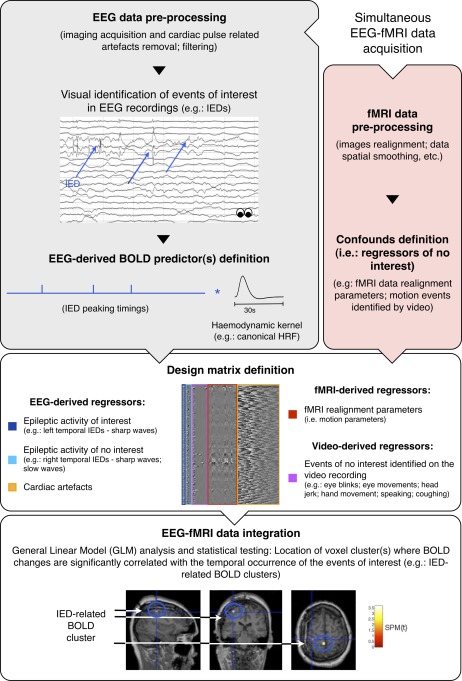
Common pipeline of EEG‐informed fMRI in epilepsy. Functional MRI is used to map the generators of interictal activity (IEDs), visually identified in the simultaneously recorded EEG. Matrix design example borrowed with permission from Chaudhary et al., Neuroimage, 2012, 61, 1383–1393. EEG traces and BOLD fMRI maps illustrations borrowed with permission from Vulliemoz et al., Neuroimage, 2011, 54, 182–190. [Color figure can be viewed in the online issue, which is available at http://wileyonlinelibrary.com.]

Electroencephalography (EEG) and functional magnetic resonance imaging (fMRI) are commonly used noninvasive techniques of functional neuroimaging that record, at a macroscopic level, signals arising from neuronal activity. A wide range of processes and phenomena has been studied using such indirect measurements of neuronal activity, ranging from physiological cognitive functions to pathological events (e.g., spontaneous epileptic discharges). Combining EEG and fMRI signals emerges as a natural consequence of the complementarity between their temporal and spatial resolutions; the origin of their sources; and the potential capability of fMRI to locate EEG generators, while avoiding the EEG inverse problem. Simultaneous EEG–fMRI measurements combined with heuristic correlation models have resulted in important insights into generators and networks involved in epileptic activity [Chaudhary et al., 2012a; Fahoum et al., 2012; Thornton et al., 2011]. Such studies represent good examples of EEG‐informed fMRI strategies, a particular asymmetric EEG–fMRI data‐driven integration approach, where EEG captures characteristic pathological temporal patterns (e.g., epileptic spikes, seizure activity, more generally sleep stages, ongoing brain rhythms and so forth) and fMRI may help to map the generators of such activity (Fig. 1). Despite such achievements, questions remain regarding the neurophysiology underlying the blood oxygen level‐dependent (BOLD) fMRI signal [Ekstrom, 2010; Logothetis, 2008, 2010], particularly in pathology. For example, the common finding in epilepsy of BOLD signal increases and decreases related to epileptic discharges has highlighted the need for a better understanding of BOLD signal generators [Pittau et al., 2013]. Furthermore, the question ‘Which neuronal processing aspects are most closely related to the BOLD signal?’ has been extensively investigated and discussed over recent years [Ekstrom, 2010; Logothetis, 2008, 2010; Magri et al., 2012]. Due to the challenging technical aspects involved, simultaneous recordings of colocalised electrophysiological and haemodynamic fluctuations have only recently been possible in humans [Boucousis et al., 2012; Carmichael et al., 2010, 2012]. This provides the opportunity to shed light on questions such as ‘What rules govern the relationship between the electrophysiological and BOLD fluctuations?’, ‘How general are these rules?’ or ‘Do BOLD decreases have a robust electrophysiological correlate, and therefore, a potentially important neurophysiological meaning?’.

Given the comparatively better understanding of the neuronal substrate of electrophysiological signals, a better understanding of the relationship between EEG and BOLD signals should lead to important insights into the neuronal substrate of the BOLD signal. Therefore, to improve the interpretability of fMRI studies (with or without EEG), this relationship needs to be further studied. Moreover, any EEG–fMRI data integration approach relies on a more or less heuristic model incorporating information about local correlations between the electrophysiological and haemodynamic signals. Even the most complex theoretically based integration approaches rely on assumptions about these local correlations, which are embedded within a mathematical model describing the relationships between the EEG signal, the BOLD signal and the underlying neuronal activity. For instance, Babajani et al. [2005] and Valdes‐Sosa et al. [2009] proposed theoretically based integration models that relied on Logothetis' (2001) findings suggesting that local filed potentials (LFPs) were better predictors of BOLD fluctuations than single‐ or multiunit activities (SUAs or MUAs), as measured with microelectrodes implanted in the visual cortex of macaques.

In this article, we review studies whose main purpose was to improve the neurophysiological interpretation of the observed BOLD patterns. Studies of normal and epileptic activities are considered, because we aim to describe the current knowledge on the electrohaemodynamic coupling in the most common EEG–fMRI application scenarios: epilepsy; nonepileptic spontaneous activity; and cognitive, sensory and motor functions. We begin by briefly describing the current understanding of the basis of the EEG and BOLD signals in Neural Basis of EEG and BOLD signals section, while aiming to understand the EEG and fMRI data complementarity. We then focus on EEG‐informed fMRI studies (asymmetric EEG and fMRI data integration approach) using simultaneously acquired data, which is useful to investigate spontaneous phenomena and the variability of individual events. A short Simultaneity of Electrophysiological and fMRI Acquisitions section is dedicated to the description of the advantages and disadvantages of simultaneous acquisitions. We also focus on human studies, apart from a subsection on the relationship between neuronal spiking activity and the BOLD signal. Electrophysiological Correlates of the BOLD Signal section comprises a revision of EEG‐informed fMRI studies grouped as (BOLD Mapping of Electrophysiological Activity section) those using fMRI to map haemodynamic changes associated with particular EEG phenomena (EEG–fMRI mapping); and (Characterising the Local Relationship Between the Electrophysiological and BOLD Signals section) those focused on characterising the local relationship between the electrophysiological and haemodynamic signals (local EEG–fMRI coupling), for example, through systematic comparisons of EEG‐derived metrics with BOLD fluctuations. Studies in BOLD Mapping of Electrophysiological Activity section are grouped according to the type of activity being addressed: epileptic; nonepileptic spontaneous activity and cognitive, sensory and motor functions. Studies in Characterising the Local Relationship Between the Electrophysiological and BOLD Signals section are grouped according to the spatial scale of the electrophysiological recordings used: neuronal spiking activity and LFPs; and intracranial EEG (icEEG).

## NEURAL BASIS OF EEG AND BOLD SIGNALS

fBrain function relies on a causal chain of events originated at the level of synapses, the basic elements for communication between neurons. Active neurons generate time‐varying electric currents, which result from ions crossing their cellular membranes. There are two main forms of neuronal activation: the slow changes in membrane potential due to synaptic activation, which are mediated by several neurotransmitter systems—these are the postsynaptic potentials (PSP; excitatory and inhibitory: EPSP and IPSP); and the fast neuronal membrane depolarisation, which results from action potentials (Lopes da Silva, 2010).

In this section, some key points about our current understanding of the neuronal origins of EEG and BOLD signals are briefly discussed. First, we describe the origin of the cerebral electrophysiological signals, recorded at different spatial scales, and how they relate to each other. Then, we introduce the electrophysiological phenomena most often being investigated in EEG‐informed fMRI studies: rhythmic activity, event‐related activity and epileptic activity. Last, we describe the basis of the BOLD effect, and how the mechanisms underlying epileptic activity‐related BOLD changes might differ from those associated with cognitive, sensory or motor activity.

### Cerebral Electrophysiological Signals

**Figure 2:**
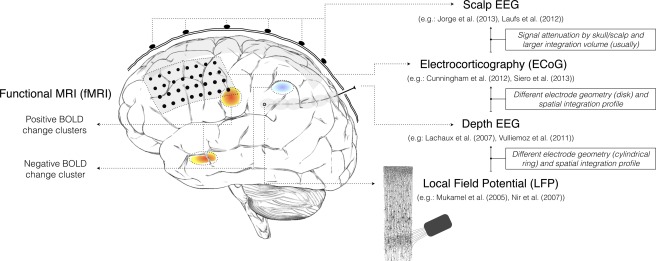
Brain signals at multiple scales: from thousands of neurons to whole‐brain. Illustration of recording of scalp EEG, ECoG, depth EEG and LFP signals, and three hypothetical BOLD‐fMRI clusters, in different lobes, on different sides of the brain. The spatial sensitivity profiles of the four electrophysiological techniques are sketched in shaded grey: mainly neocortical for scalp EEG and ECoG, local to each electrode pair for depth EEG (∼1 cm^3^), and local to each microelectrode for LFP (∼100 μm^3^). LFP recording is illustrated with a draw from Ramón y Cajal, 1899. [Color figure can be viewed in the online issue, which is available at http://wileyonlinelibrary.com.]

Electrical currents arising during synaptic activation can sum to generate electrical potentials that may be measured at different spatial scales (Fig. 2) [Kajikawa and Schroeder, 2011; Riera et al., 2005]. Most multimodal studies mapping haemodynamic changes related to specific electrophysiological phenomena use scalp EEG, whereas those focused on characterising the local relationship between the electrophysiology and BOLD often involve invasive electrophysiological recordings.

#### Recording at different spatial scales and relationship with the generators

LFPs are recorded with low‐impedance microelectrodes placed in the extracellular medium, sufficiently far from individual cells (Fig. 2) to prevent any particular neuron from dominating the signal. Action potentials (SUA or MUA, depending on the number of cells involved) are obtained by high‐pass filtering the extracellular recordings, or by placing the microelectrodes within (or close to) the cell membrane. Given the required invasiveness, LFPs, SUAs and MUAs are rarely recorded in humans.

Due to the particular geometry and hierarchical organisation of neuronal ensembles in some brain structures (e.g., cortical pyramidal cells arranged parallel to each other, with apical dendrites on one side and soma on the other), PSPs can sum into an effective current source. Given the head tissue conductor properties, this current source can be large enough to be remotely recorded (Niedermeyer and Lopes da Silva, 1999; Nunez, 1981]. Thus, scalp EEG (Fig. 2) primarily reflects the slow EPSP and IPSP of cortical populations [Creutzfeldt et al., 1966a, b; Klee et al., 1965], with some contribution from mechanisms not directly coupled to synaptic activity (e.g., voltage‐dependent membrane oscillations and spike after‐potentials) [Buzsaki and Chrobak, 1995; Creutzfeldt et al., 1966a, b; Kandel and Buzsaki, 1997; Kocsis et al., 1999].

Human icEEG is recorded for clinical purposes in patients with severe epilepsy using macroelectrode arrays (grids or strips) placed over the cortical surface (electrocorticography: ECoG) or implanted within the brain (depth EEG; or stereotactic–EEG: SEEG; Fig. 2). 1In this article, the term ‘icEEG’ is used when the statement refers to either ‘ECoG’, ‘depth EEG’ or ‘SEEG’. The more specific terms are used to inform the reader about the specific form of icEEG used in each study. Clinical icEEG has sometimes been described as a measurement of LFP [Baumgartner et al., 2011; Conner et al., 2011]; however, it represents significantly larger integration volumes than the signal recorded with microelectrodes. Due to the uncertainty in the localisation of epileptic activity generators, icEEG electrodes are frequently placed over regions that turn out to be apparently free of pathology thereby providing an opportunity to study normal brain activity [Conner et al., 2011; Hermes et al., 2012; Khursheed et al., 2011]. The availability of microelectrodes combined with the clinical icEEG electrodes is a promising research tool [Fried et al., 1999; Hochberg et al., 2006; Schevon et al., 2012; Waziri et al., 2009].

The sensitivity profile of each type of recordings is determined by the geometry and size of the electrodes. In general, icEEG has greater regional sensitivity and specificity than scalp EEG (∼1 cm^3^ for depth EEG) [Church et al., 1985]. Regarding deep generators, scalp EEG and ECoG are more affected by volume‐related averaging effects than depth EEG. However, icEEG has a limited spatial sampling due to the restricted number of electrodes that can be used, mostly due to the risks associated with the implantation procedure [Engel, 2013]. LFP and icEEG recordings provide highly regionally specific data; yet some ambiguity regarding the nature of their generating mechanism can exist, since they consist of ‘mean field signals’ that result from the collective behaviour of aggregates of neurons [Buzsaki et al., 2012]. At a larger scale, a particular distribution of electrical potentials recorded on the scalp can be explained by the activity of infinite different configurations of intracranial current sources [Fender, 1987]. EEG current sources are not simple point‐like charge accumulations, but have dipolar configurations [Bishop, 1949; Brazier, 1949; Gloor, 1983]; these are not simple dipoles, but dipole layers that are convoluted [Bishop, 1949; Gloor, 1985; Gloor et al., 1963; Vaughn, 1969, 1974]. Their particular geometry and orientation with regard to the electrodes are crucial determinants of the potential distribution within the brain or at scalp [Gloor, 1985; Gloor et al., 1963; Vaughn, 1969, 1974]. Reconstructing the current sources originating the scalp EEG measurements, so‐called the scalp EEG inverse problem, is a well‐known ill‐posed problem with no unique solution. Solving it requires prior assumptions on the number, geometry and/or location of the current sources, thereby introducing a fundamental uncertainty on the origin of the measured signals [Ferree et al., 2001]. Different inverse models are based on different a priori assumptions (for a review, see Michel et al. [2004]), and can be categorised as: (i) overdetermined (dipolar) models [e.g., Gulrajani et al., 1984; Homma et al., 1990; Kavanagh et al., 1978; Scherg and Von Cramon, 1986; Stok, 1987], based on the assumption that a small and known number of current sources can adequately model the surface measurements; or (ii) underdetermined (distributed) source models [e.g., Grave de Peralta Menendez et al., 2001; Pascual‐Marqui et al., 1994], which reconstruct the brain electric activity in each point of a three‐dimensional (3D) grid of solution points and do not need an a priori assumption on the number of current sources.

#### Important electrophysiological phenomena

As will be discussed in Simultaneity of Electrophysiological and fMRI Acquisitions section, fMRI has been used to investigate which brain regions are associated with numerous important electrophysiological phenomena described in the following.

##### Rhythmic and arrhythmic activity

The power spectra of LFP, icEEG and scalp EEG signals follow a power law distribution, that is, they may be broadly represented by a straight line on a logarithmic scale (*P*(log(*f*)) ∝ *f* ^−β^ (usually with 0 < *β* < 4)), and additional local peaks can be superimposed to it. The 1/*f* power law distribution characterises the brain arrhythmic activity, and the conspicuous peaks characterise the brain rhythms (so‐called oscillations) [Bullock et al., 2003; He et al., 2010].

These oscillatory patterns are visible in electrophysiological signals recorded either from the scalp or directly from within the brain [Buzsaki and Draguhn, 2004]; they are commonly categorised as delta (0.5–4 Hz), theta (4–8 Hz), alpha (8–12 Hz), beta (12–30 Hz) and gamma (>30 Hz) [Lopes da Silva, 2011]. The temporal characteristics of brain rhythms are phylogenetically preserved in the mammalian brain, despite their differences in size of several orders of magnitude. This preservation throughout evolution has been suggested as evidence that brain rhythms have a specific functional role (see Buzsaki et al. [2013] for further details). Furthermore, such rhythmic activity seems to be an important element linking neuronal activity and behaviour [Engel et al., 2001; Hasselmo et al., 2002; Somers and Kopell, 1993; Steriade, 2001; Traub et al., 1999; Whittington and Traub, 2003]. The terms, rhythm and oscillation, applied to electrophysiological signals are sometimes used inconsistently. Electrophysiological activity within a given frequency range does not imply that a well‐defined rhythm or oscillation exists [Lopes da Silva, 2013]. Rather, a spectral peak within the frequency band of interest must be identified, and the rhythm/oscillation is defined by the frequency, bandwidth and power of this peak. One can investigate whether a certain peak denotes a rhythm using a period specific average approach, as proposed by Bullock et al. [2003], for example. According to the latter authors, a rhythm is usually defined by a narrow peak with frequency modulation of <5% of the centre frequency, and a strength of 2.5–10 times the expectation from chance of the background noise, and shows fine structure by being local and brief (on the order of 10 cycles).

Rhythmic activity arises from competition (via inhibition) and cooperation (via excitation) occurring within local micronetworks (between individual, or small groups of, neurons) or within broad macronetworks (between large neuronal assemblies) that can exhibit different synchronisation states, resulting in oscillations at different frequencies [Pfurtscheller and Lopes da Silva, 1999; Riera et al., 2006]. The synchronisation of neuronal networks seems to enable the brain to functionally integrate computations on multiple spatial and temporal scales [Bushara et al., 2003; Buzsaki et al., 2003]. Lower oscillatory frequencies (8–12 Hz) are often associated with the recruitment of neurons from larger cortical areas. Higher oscillatory frequencies (>12 Hz) are often spatially more restricted with a functional organisation resembling a mosaic of cortical neuronal assemblies exhibiting relatively synchronous oscillations at diverse dominant frequencies [Pfurtscheller and Lopes da Silva, 1999].

Although arrhythmic activity constitutes a significant part of electrophysiological recordings, less is known about it compared to rhythmic activity [Bullock et al., 1995, 2003; Freeman and Zhai, 2009]. Interestingly, it has been suggested that synchronisation between groups of neurons may be reached not only in a rhythmic mode but also in an arrhythmic one [Eckhorn, 1994; Thivierge and Cisek, 2008].

##### Event‐related activity

A cognitive, sensory or motor stimulus can generate a time‐ and phase‐locked event‐related potential (ERP) or time‐ but not phase‐locked alterations of the ongoing EEG [Pfurtscheller and Lopes da Silva, 1999]. ERPs can be seen as a series of transient postsynaptic responses of principal pyramidal neurons, triggered by a specific stimulus [Lopes da Silva, 1991]. Assuming that the evoked activity has a fixed time delay to the stimulus and treating the ongoing activity as additive noise, ERPs are typically extracted by averaging across trials. However, intertrial variability is emerging as an important consideration when studying brain function [Debener et al., 2005; Sadaghiani and Kleinschmidt, 2013]. For instance, Fox et al. [2006] and Becker et al. [2011] found that a significant fraction of the variability of event‐related BOLD responses across trials was accounted by fluctuations in the ongoing task‐unrelated brain activity. Event‐related changes in the ongoing EEG power within specific frequency‐bands, relative to a baseline, can only be identified by a time‐frequency decomposition of individual responses. A decrease (or increase) in the synchrony of the underlying neuronal populations due to the stimulus presentation may lead to a decrease (or increase) in the ongoing power within a particular frequency‐band, so‐called an event‐related desynchronisation (ERD) [or an event‐related synchronisation (ERS); Pfurtscheller and Lopes da Silva, 1999]. ERD/ERS can be seen as reflections of the modifications in one or more parameters that regulate the neuronal networks oscillations [Lopes da Silva, 1991].

##### Epileptiform EEG activity

The EEG recordings of patients with epilepsy typically contain pathological activity during and between seizures. The power–frequency profile of the scalp EEG recorded during seizures (ictal EEG) is highly variable across patients. In focal epilepsy, the EEG at seizure onset often consists of fast and low‐amplitude activity that may spread and become slower and higher in amplitude, which reflects excessive and hypersynchronous neuronal activity [Blume et al., 2001]. In patients with severe drug‐resistant epilepsies that are undergoing presurgical evaluation, the main clinical aim of imaging and electrophysiological studies is to accurately identify the spatial contours of the brain region(s) active at the seizure onset.

The archetypal form of interictal epileptiform discharges (IED) are high‐amplitude fast EEG transients (called spikes) lasting less than 70 ms, and sharp waves lasting between 70 and 120 ms. Both spikes and sharp waves are often followed by a slow wave that can last several hundred of milliseconds [De Curtis and Avanzini, 2001]. The brain region generating interictal spikes is an important and clinically relevant marker in patients with severe drug‐resistant epilepsy. Interictal spikes tend to occur periodically [Chatrian et al., 1964] and, often, in brief paroxysms, which either remain localised in space or establish a secondary propagation to other parts of the cortex. Interictal spikes reflect the synchronous and excessive discharge of a cortical neuron ensemble and are associated with a burst discharge, which is characterised by a rapid sequence of fast action potentials at 200–500 Hz, superimposed on a slow depolarising potential [De Curtis and Avanzini, 2001].

### fMRI: The BOLD Signal

The concept of fMRI encompasses a number of MR imaging techniques capable of mapping changes in signal intensity related to changes in brain state, tasks or stimuli, reflecting changes in blood flow. The BOLD effect, which exploits an intrinsic contrast mechanism, combined with gradient‐echo Echo Planar Imaging (EPI) scanning sequences is by far the dominant contrast mechanism used for cognitive and clinical fMRI studies, at the conventional field strengths (1.5T and 3T).

#### The origin of the BOLD signal

In the current standard model of the BOLD effect, an increase in neuronal activity induces a regional increase in cerebral blood flow (CBF), which provides more oxygen and glucose to tissues and is autoregulated by local mechanisms. If the CBF increase is enough to compensate the concurrent cerebral metabolic rate of oxygen (CMRO_2_) increase [Fox and Raichle, 1986; Fox et al., 1988], the local concentration of deoxyhaemoglobin declines and the BOLD signal intensity increases [Kwong et al., 1992; Ogawa et al., 1990]. Accompanying the increase in CBF, there is an increase in the cerebral blood volume (CBV), which was characterised by Grubb et al. [1974] as cCBV(cCBF) = cCBF^α^, where cCBV and cCBF are the CBV and CBF normalised to their baseline values and α < 1. This relationship also appears in the current standard model of the BOLD effect, usually with the assumption that the fractional change in the venous CBV corresponds to the fractional change in the total CBV because most of the deoxyhaemoglobin is comprised within the venules and veins [Buxton, 2012]. However, the BOLD effect also depends on volume exchange effects that are not directly related to changes in the blood oxygenation (e.g., arterial CBV changes that displace extravascular tissue), and these effects grow with field strength [Buxton, 2012; Uludag et al., 2009].

The BOLD effect is a complex function of changes in CBV, CBF and CMRO_2_, and an important effort has been done to disentangle how changes in CBF relate to changes in CMRO_2_, which is believed to be the primary source of the BOLD effect. There is now good evidence to think of a ‘haemodynamic response’ and a ‘metabolic response’ as two independent features driven in parallel, and possibly by different aspects of neural activity [Buxton, 2012]. On the one hand, CMRO_2_ increases during the recovery from neuronal signalling (information transfer from the external environment to neurons, and conversely). For example, CMRO_2_ increases to restore ion gradients and to recycle neurotransmitters [Attwell and Laughlin, 2001; Buxton, 2012]. CMRO_2_ can reflect the overall energy cost of neuronal activity. On the other hand, there is good evidence that CBF increases may not be initiated by signals reflecting an energy deficit but instead driven by fast glutamate‐mediated signalling processes locally or by amine‐ and acetylcholine‐mediated neural systems more globally [Attwell and Iadecola, 2002]; therefore, CBF seems to be driven in a feed‐forward way by aspects of neuronal signalling [Uludag et al., 2004]. This mechanism is likely to be mediated by astrocytes acting as intermediaries between neuronal activity and blood flow, and neuronal signalling molecules (e.g., nitric oxide) acting on blood vessels diameter [Attwell and Iadecola, 2002; Buxton, 2012; Hamel, 2006; Iadecola and Nedergaard, 2007; Koehler et al., 2009]. A more detailed and complete understanding of the aspects of neural activity that strongly modulate the vascular and metabolic responses, as well as of how these responses relate to each other, will help to better describe these complex neurovascular coupling mechanisms, which link the neuronal activity to the BOLD signal.

#### Neurovascular coupling in the diseased brain

Disease may have an effect on neurovascular coupling, with important potential implications for the sensitivity and interpretation of EEG‐correlated fMRI. For example, it has been noted that the occurrence of seizures may lead to the deterioration of the cerebral glycolytic metabolic state [Folbergrova et al., 1981]; and that normal physiological CBF changes may not be sufficient to satisfy the increased metabolic demand during ictal and interictal discharges [Schwartz, 2007]. Furthermore, some mediators of the neurovascular coupling mechanisms are known to be involved in epileptogenesis [Salek‐Haddadi et al., 2003]. For instance, extracellular K^+^, which has an effect on the arteriolar diameter [Kuschinsky et al., 1972] and on CBF fluctuations [Dreier et al., 1995], rises significantly following ictal and interictal bursting [Jensen and Yaari, 1997]; and astrocytes, which play a relevant role in neurovascular coupling mechanisms [Ekstrom, 2010], are known to be an important mediator in the genesis of epileptic activity [Grisar et al., 1999]

## SIMULTANEITY OF ELECTROPHYSIOLOGICAL AND fMRI ACQUISITIONS

Single‐modality recordings performed nonsimultaneously (i.e., in different sessions) can be subsequently combined (e.g., coregistered in space, comparison of signal features and so forth), when the events of interest are considered to be reproducible across sessions. However, simultaneous sessions are essential in the following situations: (i) unique, unpredictable or uncontrolled events (e.g., epileptic activity and healthy wakeful resting); (ii) events that can only be identified or characterised on one of the modalities; (iii) individual event parameterisation (e.g., epileptic activity) and (iv) in the presence of interevent/intersession variability (e.g., habituation effects or plasticity or uncontrolled variations in response to stimulation paradigms while performing a task) [Villringer et al., 2010]. In nonsimultaneous acquisitions, the EEG recording conditions will always, to some degree, differ from those during fMRI scanning (intersession effects), even when the signals are recorded under the same external stimulus. Therefore, strictly speaking, only simultaneously acquired multimodal observations are guaranteed to relate to the same neuronal phenomenon. Nevertheless, the potential benefits of simultaneous recordings must be weighed against the associated technical difficulties: higher costs (e.g., nonparamagnetic and RF‐shielded EEG recording equipment for MR compatibility); instrumental interactions that lead to data quality degradation (e.g., MR‐environment‐related artefacts affecting the EEG; fMRI signal dropout, image distortion and nonphysiological fMRI signal changes in the electrodes vicinity; see following subsections for further details on mechanisms) [Krakow et al., 2000; Mullinger et al., 2008]; and additional safety concerns [Lemieux et al., 1997]. Depending on the phenomena of interest and the nature of the scientific question at hand, nonsimultaneous observations can be preferable [Conner et al., 2011; Hermes et al., 2012; Khursheed et al., 2011] or even necessary (such as the validation of noninvasive source localisation methods in epilepsy [Grouiller et al., 2011; Thornton et al., 2010b]).

### EEG Data Degradation Mechanisms

The major physical effect underlying the MR‐environment‐related EEG data degradation is captured by Faraday's induction law, stating that an electromotive force is induced in a conducting circuit (in this case, formed by the EEG wires, electrodes, patient and EEG amplification system) when the magnetic flux through a surface, bounded by the circuit, changes in time. Such changes in flux can be produced by the application of time‐varying magnetic fields or by movement of the conducting circuit in a static magnetic field due to, for example, the subject's head motion or to system vibrations.

Time‐varying magnetic field gradients, used for the MR signal spatial encoding, produce large changes in the magnetic flux over short time‐periods, creating voltages at the amplifier inputs that appear as artefacts one to three orders of magnitude larger than the EEG signal [Allen et al., 2000]. Such artefacts represent the most significant effect corrupting the EEG data simultaneously acquired with fMRI [Mullinger and Bowtell, 2011]. Given the repetitive nature of the gradient switching sequence, these artefacts have a strong deterministic component, based on which they can be removed [Allen et al., 2000]. However, changes in the conductive circuits geometry or position relative to the magnetic field, due to head movements or system vibrations, combined with the gradients temporal variation, can lead to random fluctuations in the induced voltages, which represent a real challenge for the artefact correction [Ritter et al., 2010].

The second most significant effect corrupting EEG data is the pulse artefact, which results from the quasi‐periodic motion and blood flow (Hall effect) linked to the cardiac cycle [Ives et al., 1993; Mullinger and Bowtell, 2011]. Because its temporal and spatial characteristics are similar across repeated cardiac cycles, a commonly used pulse artefact correction method involves the computation of an artefact template by averaging the waveform at each lead across multiple cardiac cycles [Allen et al., 2000]. However, in general, this correction is more problematic than the gradient artefact correction given the variability of the artefact waveform over time and space [Mullinger and Bowtell, 2011]. Moreover, the amplitude and spatial variability of this artefact increase with the strength of the static magnetic field, making its correction even more difficult at higher fields [Debener et al., 2008; Neuner et al., 2013].

To limit both the pulse‐ and MR acquisition‐related artefacts, useful data acquisition strategies such as limiting the area inside the conductive circuit, twisting the wires and mechanically restricting the motion of the head (e.g., vacuum cushion), have been proposed [Mullinger and Bowtell, 2011]. Even after gradient and pulse artefacts correction, EEG data may remain contaminated, especially when there is motion during the acquisition. Therefore, the identification and/or quantification of EEG phenomena may be compromised. For example, inappropriate pulse artefact correction may lead to spurious delta/theta activity, while inappropriate gradient artefact correction may lead to spurious high‐frequency activity.

### MRI Data Degradation Mechanisms

fMRI data degradation caused by the presence of the EEG recording system conducting wires and electrodes is a relatively minor effect when compared to the degradation of scalp EEG data, at simultaneous acquisitions [Mullinger and Bowtell, 2011]. It results from magnetic susceptibility effects, which lead to signal dropout and geometric distortion, as well as from the perturbation of the radio‐frequency fields, which may cause local signal changes and a global reduction in the signal‐to‐noise ratio. The impact of such effects on fMRI data quality depends on the strength of the magnetic field and the number of electrodes [Mullinger et al., 2008].

## ELECTROPHYSIOLOGICAL CORRELATES OF THE BOLD SIGNAL

**Figure 3:**
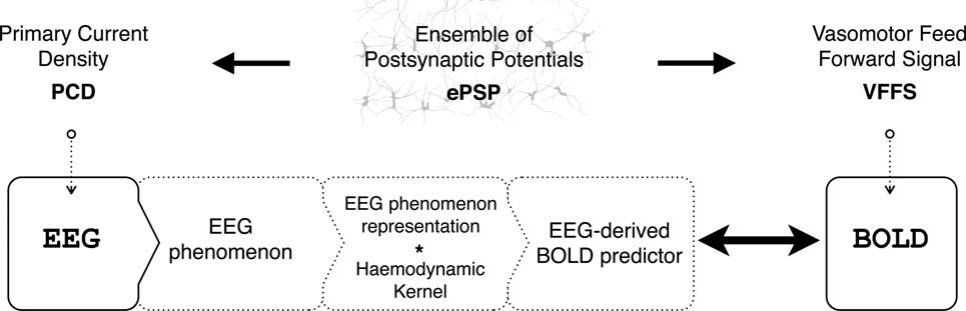
General EEG‐informed fMRI integration scheme, highlighting the mechanisms underlying each signal. In a given voxel, the neuronal activity generates an ensemble of postsynaptic potentials (ePSP). The temporarily and spatially synchronised summated PSPs produce the primary current sources (PCD). The head volume conductor properties transform the PCD into EEG. The ePSP generates a vasomotor feed forward signal (VFFS), via its own forward model, which is in turn transformed, via the haemodynamic forward model, into the observed BOLD signal. ePSP, PCD, VFFS, EEG and BOLD are time‐dependent. The EEG is considered to have the same time evolution as the PCD, which is considered to be a driver for the BOLD signal. This is an asymmetrical EEG and fMRI data integration approach because the EEG temporal dynamics are taken as surrogates for the VFFS. Diagram was adapted with permission from Valdes‐Sosa et al., Human Brain Mapping, 2009, 30, 2701–2721.

In this section, we review asymmetric EEG and fMRI data integration studies aiming to improve the neurophysiological interpretation of observed BOLD patterns. Most of these studies are based on simultaneously acquired multimodal (EEG and fMRI) data. These multimodal data integration studies may be called EEG‐informed fMRI, EEG‐correlated fMRI or simply EEG–fMRI because the EEG temporal dynamics is taken as surrogate for haemodynamic fluctuations (Fig. 3).

### BOLD Mapping of Electrophysiological Activity

In this section, we review EEG‐informed fMRI studies aiming to map the generators of different types of electrophysiological activity (e.g., spontaneous brain rhythms or epileptic activity), using simultaneously acquired EEG and fMRI data. In the general linear modelling (GLM) framework, a mathematical representation of each electrophysiological‐derived event is convolved with a haemodynamic kernel, commonly the canonical haemodynamic response function (HRF) [Logothetis, 2003], resulting in a regressor of an effect of interest [Worsley and Friston, 1995]. Statistical inferences on the estimated GLM parameters are then made to find the voxels at which the BOLD signal fluctuations are significantly correlated with the EEG‐derived regressor. EEG phenomena can be categorised as either: isolated events with varying durations (e.g., epileptic spikes, epileptic seizures, ERP amplitude and/or latency, EEG band power change time‐locked to a stimulus or task); or continuously varying (e.g., spontaneous fluctuations in EEG alpha‐band (8–12 Hz) power during awake rest, gamma‐band power fluctuations while performing a task). Studies are grouped according to the type of brain activity being addressed: epileptic; nonepileptic spontaneous activity and cognitive, sensory and motor functions.

#### Epilepsy

Given the clinical interest in spatially mapping the generators of IEDs, EEG‐informed fMRI was first implemented in the field of epilepsy [Ives et al., 1993]. fMRI held promise to circumvent the limitations imposed by the EEG inverse problem, which is particularly challenging for deep and extensive epileptic activity generators [Chaudhary et al., 2012a; Jacobs et al., 2008b; Salek‐Haddadi et al., 2006; Thornton et al., 2010a, 2011; Zijlmans et al., 2007]. For this application, the simultaneity of both signals is necessary 2This is in contrast to fMRI of epileptic seizures, which can, in some patients, be performed without simultaneous EEG, if one uses observation of the seizure clinical manifestations to inform the fMRI model. because IEDs are unpredictable and only observable on EEG [Ives et al., 1993]. Most EEG‐informed fMRI studies in epilepsy start with the identification of clinically relevant events on EEG recordings either visually by an electroencephalographer [An et al., 2013; Flanagan et al., 2014; Moeller et al., 2013; Pittau et al., 2013] or using automated signal analysis techniques [Formaggio et al., 2011; Liston et al., 2006; Marques et al., 2009]. Investigations of BOLD fluctuations related to ictal event (seizures) [Chaudhary et al., 2012a; LeVan et al., 2010b; Murta et al., 2012; Thornton et al., 2010b; Tyvaert et al., 2009; Vaudano et al., 2012] are fewer than interictal studies due to methodological and practical concerns (patient safety, relative paucity of events and seizure‐related motion; see Chaudhary et al. [2013] for a recent review). Despite both being markers of epileptogenicity, ictal and interictal discharges are most likely to be generated by different neuronal populations, through different cellular and network mechanisms [De Curtis and Avanzini, 2001]. Consequently, their BOLD correlates may well have a different physiological meaning.

##### Mathematical representation of epileptic activity for fMRI modelling

In the GLM framework, IEDs are commonly represented as (zero‐duration) stick functions at the time of maximum amplitude [Al‐Asmi et al., 2003; Salek‐Haddadi et al., 2006] or at a series of regularly spaced intervals on either side of the IED peaking time [Bagshaw et al., 2005; Jacobs et al., 2009], the latter allowing for some variation in the haemodynamic latency. IEDs with different electroclinical characteristics have been modelled as separate effects, reflecting the modelling assumption of distinct generators [Jacobs et al., 2009; Vulliemoz et al., 2010]. More complex IED‐representations have also been investigated [Formaggio et al., 2011; Leite et al., 2013; Vulliemoz et al., 2010]. For example, Vulliemoz et al. [2010] used EEG source imaging (ESI) to locate different IED sources, whose current density time course was convolved with the canonical HRF yielding to a continuous ESI (cESI) regressor. They found that cESI regressors explained additional BOLD variance in clinically expected regions, when compared to the standard regressor. Formaggio et al. [2011] used an independent component analysis (ICA) on EEG data to find potential IED generators, which were back‐projected to the EEG channels space to obtain the IED‐related EEG signals (IED–EEG). Assuming that scalp EEG signals represent a linear mixture of neuronal activities originated from independent sources, ICA can be used to unmix the signal into statistically independent subcomponents [Makeig et al., 2002]. The EEG channel exhibiting the highest correlation with the IED–EEG time course was selected, and its maximum power values within 3.7 s epochs were used to define the IED‐representative regressor, which revealed BOLD changes in clinically expected regions.

Ictal events are often represented as boxcar functions with the duration of the clinical event [Iannetti et al., 2002; Marrosu et al., 2009; Morocz et al., 2003; Salek‐Haddadi et al., 2002, 2009; Tyvaert et al., 2008]. Arguing that such an approach does not account for ictal dynamics [Niedermeyer and Lopes da Silva, 1999], more complex models of ictal BOLD changes have been proposed [Donaire et al., 2009; Thornton et al., 2010b; Tyvaert et al., 2009]. For example, Donaire et al. [2009] divided the preictal, ictal and postictal periods into a sequence of 10 s boxcar functions. Tyvaert et al. [2009] used sequential full‐width at half‐maximum 2 s gamma functions peaking around the ictal EEG onset as regressors of interest, in independent GLMs. Donaire et al. [2009] and Tyvaert et al. [2009] found a good agreement between the location of the earliest positive BOLD changes and the clinically inferred seizure onset zone (SOZ). Thornton et al. [2010b] partitioned each seizure into up to three phases, namely early ictal (first EEG changes), clinical onset and late ictal (onset of high amplitude low frequency), each represented by a boxcar function. The group found greater concordance between the most significant BOLD clusters and the SOZ (defined by icEEG), for the early ictal phase, which is not surprising because the ictal activity is likely to start at the SOZ. Leite et al. [2013] compared a number of EEG‐derived metrics representing different EEG–BOLD coupling functions (previously applied in a visual task context (Rosa et al., [2010]; see Table 1), associated with interictal and ictal activity, in a patient with a hypothalamic hamartoma. ICA was applied to EEG data, and the independent components (ICs) with mixing weight topographies in agreement with the patient's clinical history were time‐frequency decomposed. The group found that frequency‐weighted metrics yielded higher number of voxels exhibiting statistically significant BOLD changes, when compared to power‐weighted metrics or to the conventional boxcar‐based regression, in line with the heuristic proposed by Kilner et al. [2005].

**Table 1:** Heuristic EEG–BOLD transfer functions

Transfer function	Equation	Definition of terms
Total EEG power [Wan et al., 2006]	$q_{T}(t)=\sum_{f_{\min}}^{f_{\max}}P(f,t)$	*P*(*f*, *t*) = EEG power
Linear combination of EEG power over bands [Goense and Logothetis, 2008]	$q_{L}(t)=\sum_{b_{\min}}^{b_{\max}}\beta_{b}q_{b}(t)$	*q*_b_ = EEG power in band *b β*_b_ = band weight
Root mean squared frequency‐weighted EEG power [Rosa et al., 2010]	$q_{\text{RMSF}}(t)=\sqrt{\sum_{f_{\min}}^{f_{\max}}f^{2}P(f,t)}$	*P*(*f*, *t*) = EEG power
Root mean squared frequency‐weighted normalised EEG power [Kilner et al., 2005]	$q_{\text{RMSF}N}(t)=\sqrt{\sum_{f_{\min}}^{f_{\max}}f^{2}\tilde{P}(f,t)}$	$\tilde{P}(f,t)=\frac{P(f,t)}{\sum_{{f^{\prime }}_{\min}}^{{f^{\prime }}_{\max}}P(f^{\prime },t)}$
Mean squared frequency‐weighted normalised EEG power [Rosa et al., 2010]	$q_{\text{MSF}}(t)=\sum_{f_{\min}}^{f_{\max}}f^{2}\tilde{P}(f,t)$	
Mean frequency‐weighted EEG power [Rosa et al., 2010]	$q_{\text{MF}}(t)=\sum_{f_{\min}}^{f_{\max}}f\tilde{P}(f,t)$	

In summary, different representations of interictal (and ictal) activity have been related to BOLD changes within regions known to be affected; more sophisticated representations have been leading to better results [Leite et al., 2013; Vulliemoz et al., 2010], but no study so far has identified an optimal approach conclusively, in large part due to the limitations of the ‘gold standard’. For example, electroclinical information derived from scalp EEG has limited sensitivity and icEEG has limited spatial sampling. A potentially more promising approach is to compare the BOLD maps with the area of resection, in light of surgical outcome assessed in terms of seizure reduction [Thornton et al., 2010a; van Houdt et al., 2013]. However, this approach suffers from two limitations: first, seizure disruption or cessation may result from disruption of only part of the seizure generation network, making the assessment of spatial concordance complex; second, the patterns revealed by fMRI may represent a combination of epileptogenic and propagation regions. Distinguishing such regions has been attempted using diffusion tensor imaging [Hamandi et al., 2008], ESI [Vulliemoz et al., 2010] and DCM [Murta et al., 2012], for example, with some success. Further work in this area is crucial for the clinical interpretability of fMRI in epilepsy.

##### Variability of the HRF for epileptic activity

Various studies reported morphological differences in the HRF associated with epileptic discharges, when compared to that observed for sensory and cognitive events in the healthy brain [Grouiller et al., 2010; Hawco et al., 2007; Jacobs et al., 2009; Lu et al., 2006; Masterton et al., 2010; Moeller et al., 2008]. Differences across subjects [Benar et al., 2002; Grouiller et al., 2010; Kang et al., 2003], brain regions [Benar et al., 2002; Grouiller et al., 2010], age [Jacobs et al., 2008a] and discharge frequency [Jacobs et al., 2008a] have also been reported. Such findings do not necessarily imply a systematically abnormal HRF, since significant variability has also been reported in healthy subjects [Aguirre et al., 1998; Handwerker et al., 2004; Miezin et al., 2000]. In fact, such findings only report that different HRFs are estimated from data in different conditions, when particular EEG–fMRI integration models are assumed (which can be close/far from the real physiology; and which imply the choice of particular EEG‐phenomena (recall Important Electrophysiological Phenomena subsection). The few studies in focal epilepsy assessing the statistical significance of such deviations within individual subjects have found weak effects [Lemieux et al., 2008]. Nevertheless, it is worth mentioning the work by van Houdt et al. [2013] that estimated the shape of the HRF within the SOZ in a group of patients. A canonical shape was found in some cases, but not in others, which indicates that the use of a standard HRF model may lead to false‐negative fMRI results. One of the most striking patterns of HRF deviation from the norm is, perhaps, that observed in generalised epileptic activity: BOLD changes that seem to precede epileptic discharges (assuming a normal haemodynamic delay) [Jacobs et al., 2009], which may reflect neural activity not observed on scalp EEG, but time locked to the epileptiform discharges.

In general, finding morphological differences in HRFs for epileptic discharges may result from different factors: an altered electrohaemodynamic coupling [LeVan et al., 2010a; van Houdt et al., 2013]; differences in neuronal response timings (faster HRFs related to neuronal activity at the epileptic focus; slower HRFs related to propagated activity) [Kobayashi et al., 2009; Lemieux et al., 2008; LeVan et al., 2010b]; or spatially nonoverlapping EEG and BOLD signals [Disbrow et al., 2000; Jacobs et al., 2009; Laufs, 2008]. Using simultaneously acquired icEEG–fMRI data and assuming a particular EEG‐derived representation of epileptic activity, we will be able to further explore the morphology of HRFs derived from the SOZ and other regions exhibiting propagated activity, being aware of the spatial overlap between the signals. However, it will still be hard to distinguish between an altered electrohaemodynamic coupling and an inadequate EEG‐derived representation of brain activity.

##### Negatively correlated BOLD and epileptic discharges

Regional BOLD changes negatively correlated with epileptic activity have been observed in focal and generalised epilepsies [Benar et al., 2006; Gotman et al., 2006; Grouiller et al., 2010; Lemieux et al., 2008; Moeller et al., 2009; Salek‐Haddadi et al., 2006]. In generalised epilepsies, commonly widespread spike‐related BOLD decreases have been detected in the cortex [Gotman et al., 2005; Hamandi et al., 2006; Salek‐Haddadi et al., 2003]. Carmichael et al. [2008] confirmed (through arterial spin labelling) that CBF and BOLD signals remained normally coupled (i.e., positively correlated) in regions exhibiting strong decreases. Epileptic spike‐related BOLD decreases remain unexplained because epileptic discharges are a marker of neuronal hyperexcitability, while BOLD decreases are thought to mainly reflect decreased blood flow linked to decreased neuronal activity [Shmuel et al., 2006] (although purely haemodynamic effects have also been proposed [Harel et al., 2002]). Using fMRI, CBV, CBF, neuronal recordings and CMRO_2_ modelling, Schridde et al. [2008] argued that a sustained BOLD decrease does not unequivocally imply decreased neuronal activity or CBF. In fact, it can also result from increased neuronal activity depending on the complex interplay between haemodynamics and metabolism (e.g., if CMRO_2_ increase during seizures nearly matches CBF increase, suggesting that oxygen consumption might at certain times exceed its supply, a weak or even decreased BOLD signal due to increased deoxygenated haemoglobin content is observed, despite strong increased neuronal activity). Schridde et al. [2008] concluded that BOLD decreases might result from an increase or decrease in neuronal activity, depending on the brain state and region.

In summary, a number of explanations for epilepsy‐related BOLD decreases have been offered, which may be explored further using simultaneous icEEG–fMRI data recorded in humans, with its exquisite local sensitivity [Cunningham et al., 2012; Vulliemoz et al., 2011].

##### Simultaneous icEEG–fMRI in epilepsy

Vulliemoz et al. [2011] and Cunningham et al. [2012] have recently mapped IED‐related BOLD changes using simultaneous icEEG–fMRI. Vulliemoz et al. [2011] investigated two patients whose scalp EEG–fMRI recordings (performed prior to the icEEG implantation) did not show IEDs. Nevertheless, the icEEG–fMRI investigation revealed IED‐related BOLD changes, both in regions close to the most active icEEG contacts and remote regions. In one case, an epileptic network including regions that could not be sampled by icEEG was found, in agreement with previous MEG investigations. In this case, the persistence of seizures after the resective surgery suggested that such regions (invisible to icEEG) were likely to have key roles within the network. Cunningham et al. [2012] also investigated two patients and found IED‐related BOLD changes at locations in broad agreement with noninvasive findings. In one patient, some remote BOLD clusters matched regions of hyperperfusion revealed by ictal single‐photon emission computerized tomography (SPECT). Furthermore, both groups found IED‐related BOLD decreases in the SOZ, in a few patients. Vulliemoz et al. [2011] and Cunningham et al. [2012] have shown that acquiring simultaneous icEEG–fMRI data at 1.5T and 3T is feasible, in humans, under certain conditions; and that such technique offers not only the measurement of haemodynamic responses over the whole brain but also the unsurpassed regional specificity and sensitivity of icEEG.

#### Nonepileptic spontaneous activity

EEG‐informed fMRI studies on nonepileptic forms of spontaneous cerebral activity have also been performed. Most of them investigate the spatial distribution of positive and negative correlations between the power within the classical EEG frequency‐bands and the BOLD signal [Laufs et al., 2003b; Martinez‐Montes et al., 2004].

Selecting one or some meaningful EEG features, that is, representations of EEG phenomena of interest, is often the first challenge in EEG‐informed fMRI studies (Fig. 3) during rest. Visual inspection and channel selection or, instead, recurring to blind signal separation algorithms, are examples of strategies often used to identify such features. For example, Goldman et al. [2002] selected four occipital bipolar channels because they were particularly interested on the posterior dominant alpha rhythm (in contrast to other rhythms in the same frequency range). Scheeringa et al. [2008], for instance, performed temporal ICA on band‐pass filtered (2–9 Hz) scalp EEG data, and selected the IC with mixing weights topography with a midfrontal topography because they were particularly interested in the frontal theta. Most EEG‐derived features during rest consist of the ongoing EEG power within one (or more) classical frequency‐band, which is convolved with the HRF to obtain a regressor of interest to include in a GLM of BOLD changes.

##### Mapping the occipital alpha rhythm during rest

From the early days of EEG‐informed fMRI, a considerable interest in investigating the spatial distribution of statistically significant correlations between the occipital ongoing scalp EEG alpha power and BOLD fluctuations has been noted [De Munck et al., 2007; Difrancesco et al., 2008; Goldman et al., 2002; Goncalves et al., 2006; Laufs et al., 2003a, b; Moosmann et al., 2003]. Negative correlations were found in occipital [De Munck et al., 2007; Difrancesco et al., 2008; Goldman et al., 2002; Goncalves et al., 2006; Moosmann et al., 2003], temporal [Goldman et al., 2002], parietal [De Munck et al., 2007; Goncalves et al., 2006; Laufs et al., 2003a, b] and frontal [Goldman et al., 2002; Goncalves et al., 2006; Laufs et al., 2003a, b] cortices. Positive, locally restricted, correlations were found in the thalamus [De Munck et al., 2007; Difrancesco et al., 2008; Goldman et al., 2002; Goncalves et al., 2006]. Laufs et al. [2003a], De Munck et al. [2006] and Goncalves et al. [2006] observed that such correlation patterns did not depend greatly on the selected EEG channels, but their statistical significance was higher when only occipital channels were used. Martinez‐Montes et al. [2004] used a less conventional method (multiway partial least‐squares analysis) to decompose both EEG (independent variable) and fMRI data (dependent variable) uniquely as a sum of ‘atoms’ (each EEG atom is the outer product of spatial, spectral and temporal signatures; each fMRI atom is the product of spatial and temporal signatures). In agreement with other studies, the group found positive focal correlations in thalamus. Notably, Laufs et al. [2003a, b] did not find these correlations. Moosmann et al. [2003] argued that such correlations may be of artefactual origin due to cardiac pulse effects in the nearby ventricles. If not of artefactual origin, these findings are relevant because invasive studies in animals proposed that the thalamus has a central role in the generation and modulation of cortical alpha rhythm, and that its activity has an important relationship with the neocortical rhythmic activity [Lopes da Silva et al., 1973].

More recently, Jann et al. [2009] investigated the relationship between the alpha rhythm global field synchronisation (GFS; a measure of the zero‐phase lag synchronisation between electrodes) [Koenig et al., 2001, 2005] and BOLD fluctuations during rest, with closed eyes. Regarding the lower alpha (8.5–10.5 Hz) GFS: positive correlations were found in the anterior and posterior cingulate cortices, and in the orbitofrontal and parietotemporal regions; negative correlations were found in the superior frontal gyrus, insula, supramarginal gyrus and supplementary motor areas, which is a group of regions previously identified as constituting a brain network, the default mode network (DMN) [Raichle et al., 2001; Raichle and Mintun, 2006]. The DMN is an example of a resting state network (RSN) and its function has been the subject of numerous investigations. It has been observed that the DMN activity is greater during rest, compared with states of reduced consciousness or extroverted perception and action [Mazoyer et al., 2001]. In general, RSNs are synchronised fluctuating networks that involve cortical and subcortical areas, which can be obtained by spatial ICA of the resting state BOLD time courses across the brain [Beckmann and Smith, 2004]. These independent varying patterns of signal coherence arise from the resting brain, involve cortical areas and show similar spatial configurations to functional–anatomical networks, usually recruited by specific cognitive processes, suggesting that RSNs represent the ‘default’ state of such functional–anatomical networks [De Luca et al., 2006]. Jann et al. [2009] also found a positive correlation between the upper alpha (10.5–12.5 Hz) GFS and BOLD fluctuations in the dorsal attention network (DAN) RSN, which included a posterior portion of the cingulate gyrus, bilateral dorsolateral prefrontal cortex (DLPFC) and areas in the parietal lobe.

##### Mapping other EEG frequency components during rest

Aiming to investigate a broader EEG frequency content, Laufs et al. [2003b] included the occipital theta, alpha and beta powers within a single GLM. No significant correlations were found for the occipital theta (4–7 Hz) power. Positive correlations in the DMN [Raichle et al., 2001] were found for the beta (17–23 Hz) power. A few years later, Mantini et al. [2007] investigated the correlation between the delta, theta (4–8 Hz), alpha (8–13 Hz), beta (13–30 Hz) and gamma (30–50 Hz) powers, averaged across the scalp, and BOLD changes within six RSNs and found that each RSN was characterised by a specific electrophysiological signature, reflecting a specific combination of EEG frequency components. More recently, De Munck et al. [2009] included all the classical EEG frequency components in a single GLM and showed that the different EEG frequency components were mutually correlated. The authors suggest that the brain does not generally display pure frequency components within distinct bands, mostly generated in restricted neuronal circuits, but a coalescence of these different frequency components, and their interactions must be accounted for [De Munck et al., 2009] and concluded that all bands should be modelled in this type of study, irrespective of the specific band of interest.

In summary, the occipital alpha rhythm power was negatively correlated with widespread occipital, parietal, temporal and frontal BOLD changes and it seemed to be positively correlated with a further remote (far from scalp electrodes) brain area, the thalamus. Probably due to practical and historical reasons, the most explored electrophysiological feature has been the alpha rhythm. However, the BOLD correlates of other EEG frequency components are potentially as interesting as the alpha ones, as will become apparent in a few paragraphs ahead.

##### BOLD fMRI functional connectivity and the EEG

The growing interest in fMRI functional connectivity has led to studies on its EEG correlates [Chang et al., 2013; Scheeringa et al., 2012; Tagliazucchi et al., 2012]. Scheeringa et al. [2012] explored the relationship between posterior alpha power and inter‐regional BOLD fMRI functional connectivity during awake rest, and found that low alpha power periods were significantly associated with stronger functional connectivity between: primary visual cortex and remaining occipital cortex (positively coupled); primary visual cortex and anterior‐medial thalamus (negatively coupled); and primary visual and ventral‐medial prefrontal cortices (negatively coupled). Tagliazucchi et al. [2012] investigated how frontal, central and occipital EEG power relates to various characterisations of fMRI functional connectivity, and found significant negative correlations between alpha power and the strength of connectivity between subcortical areas, association and primary cortices; significant positive correlations between frontal gamma power and the strength of connectivity within (not between) primary, subcortical and association systems, during awake rest, as well as between frontal and central alpha power and the ‘average path length’, a graph metric that reflects the minimum number of links (between graph nodes) that have to be crossed to go from region A to B. Chang et al. [2013] investigated whether temporal fluctuations in coupling between three major RSNs (DMN, DAN and saliency network) were associated with fluctuations in alpha and theta power, during rest with eyes closed, and found that alpha power was negatively correlated with the DMN–DAN connectivity strength.

In summary, fluctuations in EEG power, within particular frequency ranges, were found to be coupled with fluctuations in BOLD functional connectivity, either local, widespread or between two RSNs, which adds another degree of complexity to the relationship between the electrophysiological and haemodynamic signals.

Another interesting subject for further investigation is how different scales of ongoing functional connectivity, such as ‘the intrinsic coupling modes’ described by Engel et al. [2013], relate to each other. Engel et al. [2013] suggested the existence of main coupling modes: one arising from phase coupling of band‐limited oscillatory signals (on the timescale of 1–1,000 ms, as measured by EEG); and another that can be described as coupled aperiodic fluctuations of signal amplitudes (on the timescale of several seconds, as measured by EEG envelopes or BOLD signal amplitudes). Using simultaneously recorded EEG–fMRI data for such purpose, while very promising, remains relatively unexplored (Bettus et al. [2011] is the only related example to our knowledge).

#### Cognitive, sensory and motor functions

Here, we review studies mapping the BOLD correlates of a few EEG phenomena during cognitive, sensory and motor functions: ERPs amplitude and latency; EEG synchronisation and phase coherence; and EEG frequency content.

##### Mapping ERP features

Several studies using simultaneous EEG–fMRI have investigated single‐trial correlations between the amplitude and/or latency of ERPs, derived from scalp EEG recordings, and the BOLD signal [Benar et al., 2007; Debener et al., 2005; Eichele et al., 2005; Fuglo et al., 2012; Mulert et al., 2008]. Mulert et al. [2008] investigated the spatial distribution of statistically significant correlations between fluctuations in the amplitude of the N1 potential (AofN1), measured during a forced choice reaction task, under low‐ and high‐effort conditions and the BOLD signal. By comparing the AofN1‐related BOLD changes under high‐effort versus passive listening, positive correlations were found in the anterior cingulate cortex (CC). The group showed that single‐trial correlations can be particularly helpful to separate different aspects of the BOLD signal based on their specific correlation to different ERP features (e.g., N1 potential fluctuations due to the high‐effort condition).

##### Mapping EEG synchronisation and phase coherence during tasks

Single‐trial scalp EEG synchronisation and phase coherence have also been investigated as potential predictors of BOLD fluctuations [Kottlow et al., 2012; Mizuhara et al., 2005]. Kottlow et al. [2012] searched for gamma (40–42 Hz) GFS‐related BOLD changes, during film viewing. The film consisted of face parts changing their positions, which, during some periods, rearranged themselves yielding a visually recognisable face (FACE). This was the first study analysing the BOLD correlates of common‐phase signals during visual binding in humans. A unitary boxcar regressor (FACE) and a scaled boxcar regressor (modulated by the GFS values; GFS–FACE) were convolved with double‐gamma HRFs. The GFS–Face regressor was orthogonalised with respect to the FACE regressor. Positive GFS–FACE–BOLD correlations were revealed in the bilateral middle fusiform gyrus and left precuneus; important areas for visual binding and face perception.

##### Mapping EEG frequency content during tasks

BOLD correlates of classical EEG frequency‐bands have also been investigated during tasks [Michels et al., 2010; Mulert et al., 2010; Scheeringa et al., 2011a, b]. Michels et al. [2010] investigated EEG–BOLD signal correlations for theta (5–7 Hz), alpha (8–10 Hz; 10–12 Hz), beta (13–20 Hz; 20–30 Hz) and gamma (30–40 Hz) power during the retention phase of a working memory task in humans. All frequency‐bands were included in the same GLM. Only positive correlations were found for high‐beta (20–30 Hz) in DLPFC and inferior frontal gyrus (IFG); and gamma in IFG and medial prefrontal cortex (MPFC). Only negative correlations were found for theta in MPFC, posterior parietal cortex, CC; and high‐alpha (10–12 Hz) in parieto‐occipital regions. Positive and negative correlations were found for low‐alpha (8–10 Hz) and low‐beta (13–20 Hz), in diverse locations. They concluded that both low and high EEG frequency‐bands correlate with the BOLD signal, in diverse locations; and that such correlations tend to be negative for lower frequencies (<7 Hz) and positive for higher ones (>20 Hz). Mulert et al. [2010] investigated the BOLD correlates of gamma (40 Hz) amplitude during an auditory task and found positive correlations in the auditory cortex, thalamus and anterior CC. Scheeringa et al. [2011a] explored how the synchronisation (zero‐phase lag) across different EEG frequency components was related with occipital BOLD fluctuations, in healthy subjects performing a visual attention task that induces sustained changes in synchronisation across a wide frequency range. They found single‐trial positive correlations between high‐gamma (60–80 Hz) power and occipital BOLD and negative single‐trial correlations between alpha (∼10 Hz) and beta (∼20 Hz) powers and occipital BOLD. Scheeringa et al. [2011b] compared the strength of the neuronal evoked response, as indexed by the BOLD signal, for visual stimuli given at the peak and the trough of the alpha cycle, and found a stronger positive BOLD response for stimuli arriving at the peak. Such findings suggest that the alpha rhythm phase at which the stimuli are given has an impact on the neuronal haemodynamic response [Scheeringa et al., 2011b].

In the above, we have reviewed studies mapping the BOLD correlates of different electrophysiologically derived quantities, over the entire brain. However, a more general question of interest might be: ‘What aspects of EEG best correlate with the BOLD signal, at any given location?’, in other words, ‘Which EEG‐derived metric best predicts focal BOLD fluctuations?’ and ‘To what degree?’. Aiming to address this question in part, Rosa et al. [2010] compared several heuristic EEG‐derived metrics in the form of moments of the EEG spectrum (in the range 1–40 Hz; Table 1), in terms of their capability to explain the occipital BOLD fluctuations recorded in healthy subjects performing a visual task. The group found that the task‐related occipital BOLD changes were best explained by the root mean squared frequency function, proposed by Kilner et al. [2005], *q*_RMSF_, which revealed more significant voxels and higher statistical significance levels.

The studies here discussed suggest that the relationship between EEG and BOLD signals is a complex function of amplitude/power, frequency and, potentially, phase of the EEG signal, in line with the resting state studies previously discussed in Nonepileptic Spontaneous Activity subsection. All these studies are somewhat unsatisfactory given the intrinsic limitations of scalp EEG, and the difficulty of defining what a better fit with BOLD is. With scalp EEG, is hard to know the location of the neural activity specifically responsible for, or associated with, the EEG and BOLD signals. In ECoG–BOLD Coupling in Humans and Depth EEG‐, LFP‐, and MUA–BOLD Coupling sections, we return to this question, at a local level, based on invasive recordings.

#### Improving the interpretation of scalp EEG‐informed fMRI

While aiming to physiologically interpret scalp EEG–fMRI mapping results, the uncertainty in our understanding of the nature of the neuronal activity being mapped is a severe limitation. For example, it is often hard to know if the BOLD fMRI technique is localising the primary generator of the EEG activity or if it is, instead, localising brain regions whose activity is a systematic consequence of the primary generator's activity (e.g., propagated epileptic activity 3The possibility of the converse of propagation (or downstream) effects, namely preparatory (or upstream) effects, is also acknowledged. For example, it is conceivable that changes in the pattern of activity in regions outside the generator itself are needed for the generator to reach the state required to generate a particular type of activity.), which is time‐locked and correlated to the first. In part, such uncertainty results from the fact that scalp EEG is mostly sensitive to neocortical activity, when compared to that of deep generators, and has limited spatial resolution. Using simultaneous icEEG–fMRI recordings while having electrodes covering not only the activity generator but also (at least part of) the propagation network, can help to distinguish the two types of activity. More importantly, the physiological interpretation of scalp EEG–fMRI mapping studies may be improved using electrophysiological features that not only (i) explain colocalised BOLD fluctuations better but also (ii) reflect well‐known aspects of the neuronal activity. Finding these more informative features can best be done using local measures of electrophysiological activity as icEEG, and further investigating the colocalised haemodynamic fluctuations.

### Characterising the Local Relationship Between the Electrophysiological and BOLD Signals

In this section, we review studies regarding the identification of the best electrophysiological‐derived predictors of the BOLD signal, helpful to improve our understanding of the BOLD origin. Most studies consist of defining alternative electrophysiologically derived quantities, and comparing the significance of the correlation between such quantities and the colocalised BOLD fluctuations. This section is divided into studies on ECoG–BOLD coupling in humans; and Depth EEG‐, LFP‐ and MUA–BOLD coupling (in both humans and nonhumans), as these correspond to two broadly distinct scales of electrophysiological integration.

#### ECoG–BOLD coupling in humans

Khursheed et al. [2011] used ECoG and fMRI data sequentially recorded from subjects performing the same working memory task. The group compared the mean ECoG power standardised difference spectra (between delay period and background; 50 periods average) between the electrodes close to and far from the task‐related BOLD changes (Statistical Parametric Mapping (SPM) maps obtained with a boxcar‐task regressor) and found that BOLD fluctuations were positively correlated with ECoG gamma (30–200 Hz) power and negatively correlated with ECoG theta (4–8 Hz) power, during the working memory delay periods. Conner et al. [2011] investigated the correlation between sequentially recorded ECoG and BOLD, while subjects performed the same visually cued noun and verb generation task. The ECoG activity within each frequency band was linearly regressed with the BOLD changes mean *t*‐value, found within regions located around each electrode. The group found positive correlations for gamma (60–120 Hz) and negative correlations for beta (13–30 Hz) ECoG bands, with gamma and beta activities independently explaining different components of the local BOLD signal. Hermes et al. [2012] studied the spatial relationship between the amplitude of task‐induced BOLD changes and the amplitude of task‐induced ECoG power changes, in the primary sensorimotor cortex. Using ECoG and fMRI data sequentially recorded during a finger movement task, they found that increases in the amplitude of gamma (65–95 Hz) power changes were colocalised with increases in the amplitude of BOLD changes, and that decreases in the amplitude of low frequency (<30 Hz) power changes were colocalised with weaker increases in the amplitude of task‐induced BOLD changes. They also found that the task‐induced fluctuations of low‐ and high‐frequency ECoG powers explain spatially distinct BOLD changes; and that, together, these spectral fluctuations account for 36% of the spatial variance of task‐induced fluctuations of the BOLD signal. Kunii et al. (2013) investigated the correlation between ECoG high‐gamma (60–120 Hz) power and BOLD fluctuations around electrodes exhibiting ECoG high‐gamma power increases due to a word interpretation task, and found that BOLD fluctuations were strongly correlated with long‐lasting high‐gamma power increases in frontal language areas, but weakly correlated with short‐duration high‐gamma power increases in temporal language areas. Siero et al. [2013] investigated how ECoG high‐gamma (65–95 Hz) power related to task‐expected and measured BOLD fluctuations in the sensorimotor cortex, during a motor task with increasing movement rates, and found that although task‐derived linear models failed to predict measured BOLD fluctuations, colocalised measured ECoG power and BOLD fluctuations were highly correlated, concluding that a large portion of the BOLD nonlinearity with respect to behaviour (movement rate) was well predicted by electrophysiology.

#### Depth EEG‐, LFP‐ and MUA–BOLD coupling

Lachaux et al. [2007] combined depth EEG and fMRI data sequentially recorded from subjects performing the same semantic decision task and found a close spatial correspondence between regions of positive BOLD changes and recording sites showing increased EEG power in the gamma range (>40 Hz). Due to the invasiveness of these recordings and associated technical difficulties, the majority of these studies were performed in animals. Logothetis et al. [2001] recorded, simultaneously, the electrophysiological (using implanted microelectrodes) and haemodynamic (using BOLD–fMRI) signals in the primary visual cortex of anaesthetised monkeys performing a visual task. The BOLD signal exhibited a strong correlation with colocalised LFPs (10–130 Hz) and a robust, but slightly weaker, correlation with colocalised MUA (300–3,000 Hz). Across recordings sites, LFPs accounted for significantly larger amounts of BOLD variance [Logothetis and Wandell, 2004]. Earlier studies in the rat cerebellum suggested that regional increases in CBF could be strongly correlated with LFPs, but also that they were present in the absence of spiking activity (manipulated with drugs) [Mathiesen et al., 1998; Mathiesen et al., 2000]. Later studies, also in animals, supported these findings [Bartolo et al., 2011; Gsell et al., 2006; Hewson‐Stoate et al., 2005; Huttunen et al., 2008; Kayser et al., 2004; Lippert et al., 2010; Martin et al., 2006; Masamoto et al., 2008; Niessing et al., 2005; Ureshi et al., 2004; Yen et al., 2011]. Mukamel et al. [2005] compared MUA and LFPs (measured using microelectrodes in a group of patients with epilepsy) with BOLD fluctuations (measured in a group of healthy subjects), exposed to the same stimulus paradigm. The group found equally good correlation between local BOLD fluctuations and LFPs and the spiking rate activity. Other studies, involving electrical stimulation of the forepaw in rats [Smith et al., 2002], or visual tasks in other animals [Kim et al., 2004; Nir et al., 2007], were consistent with such BOLD signal dependence on the neuronal spiking rate. Recent studies suggested that the relationship between the spiking rate and the BOLD signal is dependent on the site being investigated and on the stimulus paradigm being used [Lippert et al., 2010; Maier et al., 2008]. There seems to be some agreement that the BOLD signal is primarily a reflection of changes in LFPs [Goense and Logothetis, 2008]. The correlation between LFPs and spiking rates appears to be dependent on the regional input and on what neuronal circuit is being stimulated [Mitzdorf, 1985]. Therefore, depending on the conditions, the BOLD signal may actually reflect both LFPs and neuronal spiking rates [Ekstrom, 2010]. To improve the specificity of electrophysiology–BOLD correlation studies, the LFPs spectral content must be considered and the interdependence across its frequency components must be further explored.

Since different LFP frequency‐bands correlate with different behavioural states [Basar, 1980; Lindsley and Wicke, 1974; Steriade and Hobson, 1976], and seem to reflect activity of different neuronal processing pathways [Belitski et al., 2008], exploring the relationship between different LFP frequency‐bands and simultaneous haemodynamic fluctuations may lead to a more specific understanding of the BOLD signal origins. Logothetis et al. [2001], Kayser et al. [2004], Niessing et al. [2005], Nir et al. [2007], Goense and Logothetis [2008], Murayama et al. [2010] and Ojemann et al. [2010] found that local BOLD fluctuations were positively correlated with the LFP gamma power. Whether the BOLD signal independently relates to each LFP frequency‐band or a particular relationship among various LFP frequency‐bands still needs to be further investigated [Kilner et al., 2005; Magri et al., 2012]. Magri et al. [2012] found that LFP alpha and beta (18–30 Hz) powers had additional BOLD signal predictive power compared to LFP gamma (40–100 Hz) in the early visual cortex of anesthetised monkeys during spontaneous activity (darkness, eyes closed, no direct visual stimulation). In particular, they observed that an increase in LFP alpha power, without a change in the total power, was correlated with a reduction of the BOLD fluctuations amplitude, whereas an increase in the LFP gamma (40–100 Hz) power, without a change in the total power, was correlated with an augment of the BOLD fluctuations amplitude. Notably, their findings were concordant with the heuristic proposed by Kilner et al. [2005].

## CONCLUSIONS

Our review aimed to establish the state of knowledge on the relationship between electrophysiological and BOLD signals in humans, by surveying two types of studies: those using fMRI to map haemodynamic changes related to particular electrophysiological phenomena; and those focused on characterising the local relationship between the electrophysiological and BOLD signals through the identification of the electrophysiological features that best correlate with colocalised haemodynamic fluctuations. Studies using LFPs, icEEG or scalp EEG recordings during cognitive, sensory and motor functions have systematically revealed positive correlations between the power of the electrophysiological signal within the gamma ranges (>30 Hz) and the BOLD fluctuations at the same location, or at brain regions expected to be activated given the task used. Most studies also revealed negative correlations between the power of the electrophysiological signal within the low‐frequency ranges (alpha, beta and theta) and the BOLD fluctuations at the same location, or at brain regions expected to be activated given the task used. The study by Magri et al. [2012] on spontaneous brain activity in the visual cortex of anesthetised macaques is particularly important, revealing an identical profile of correlations, suggesting that the electrophysiological–haemodynamic coupling may be identical for task and nonepileptic rest conditions. Such power‐ and frequency‐based profile of correlations is still to be explored for epileptic activity (apart from a case study by Leite et al. [2013]). Almost certainly, to improve our understanding of the electrophysiological–haemodynamic coupling in all brain conditions, the complete electrophysiological spectral content should be considered and modelled. The concrete possibility of correlating icEEG measurements with simultaneously acquired BOLD fluctuations [Boucousis et al., 2012; Carmichael et al., 2010] in humans provides an excellent opportunity to further explore these relationships.

Our review further illustrates additional levels of complexity on the relationship between the electrophysiological and haemodynamic signals, by presenting and discussing studies reporting that electrophysiological signal‐derived metrics (based on spectral characteristics of the signal) are related not only with the amplitude of BOLD changes but also with measures of functional connectivity derived from the BOLD signal. These are additional aspects to be explored regarding our understanding of the electrophysiological–haemodynamic coupling.

Using simultaneous icEEG–fMRI data, a number of electrophysiological phenomena have already been studied with unprecedented sensitivity and specificity: IED‐related BOLD changes [Cunningham et al., 2012; Vulliemoz et al., 2011], beta‐band power‐related BOLD changes in the sensorimotor cortex during rest [Carmichael et al., 2011a] and different ECoG frequency components‐related BOLD changes in the motor cortex of subjects performing a finger‐tapping task [Carmichael et al., 2011b]. IcEEG signals are much more sensitive reflections of the local underlying neuronal activity, when compared to the scalp signal. Furthermore, the neuronal activity previously measured with fMRI, but inaccessible with scalp EEG, can now be recorded by both techniques. Also, icEEG signals can be colocalised with BOLD, knowing, however, that the fMRI signal dropout, close to the metallic contacts, can be a limitation [Boucousis et al., 2012; Carmichael et al., 2010]. With such enlightening data in humans, it is not only interesting to confirm the validity of the previous observations in nonsimultaneous studies but also to explore new types of electrophysiological phenomena in terms of their capability to predict the colocalised BOLD signal. For example, it would be interesting to investigate how the phase of different electrophysiological components influences the power‐ and frequency‐based profile of electrophysiological–haemodynamic correlations.

Simultaneous icEEG–fMRI data is also potentially very informative to explore and relate different scales of ongoing functional connectivity, such as those described by Engel et al. [2013] as ‘the intrinsic coupling modes’: one arising from phase coupling of band‐limited oscillatory signals (on the timescale of 1–1,000 ms, as measured by EEG); and other that can be described as coupled aperiodic fluctuations of signal amplitudes (on the timescale of several seconds, as measured by EEG envelopes or BOLD signal amplitudes). This perspective, while very promising, remains relatively unexplored (Bettus et al. [2011] is the only related example to our knowledge).

Given the current importance of fMRI in neuroscience and our need for a better understanding of the fMRI maps obtained in humans, finding a universal ‘electrophysiological–haemodynamic coupling function’, capable of accounting for a large proportion of the BOLD signal variance in different brain states (e.g., during rest, different task types and epileptic activity), would be a great advance. By allowing the investigation of local correlations between the electrophysiological and haemodynamic signals, the availability of simultaneous icEEG–fMRI data recorded in humans should provide important new insights into the relationship between the two signals and shed light on the meaning and modelling of BOLD responses.
